# New staging systems for left-sided colon cancer based on the number of retrieved and metastatic lymph nodes provide a more accurate prognosis

**DOI:** 10.3389/pore.2023.1610874

**Published:** 2023-02-24

**Authors:** Guiru Jia, Purun Lei, Yanru Zhang, Zongheng Zheng, Jiafeng Fang, Xiaofeng Yang, Hongbo Wei, Tufeng Chen

**Affiliations:** Department of Gastrointestinal Surgery, The Third Affiliated Hospital, Sun Yat-sen University, Guangzhou, China

**Keywords:** left-sided colon cancer, retrieved lymph nodes, log odds of positive lymph nodes, revised nodal stage, staging system

## Abstract

**Objectives:** We aimed to explore reasonable lymph node classification strategies for left-sided colon cancer (LCC) patients.

**Methods:** 48,425 LCC patients from 2010 to 2015 were identified in the US Surveillance, Epidemiology, and End Results database. We proposed an innovative revised nodal (rN) staging of the 8th American Joint Committee on Cancer (AJCC) Tumor/Node/Metastasis (TNM) classification based on the cut-off value of retrieved lymph nodes and survival analyses in patients with LCC. Log odds of positive lymph nodes (LODDS) stage is a numerical classification strategy obtained by a formula that incorporates the numbers of retrieved and positive lymph nodes. To develop the TrN or TLODDS classification, patients with similar survival rates were grouped by combining T and rN or LODDS stage. The TrN or TLODDS classification was further evaluated in a validation set of 12,436 LCC patients from 2016 to 2017 in the same database and a Chinese application set of 958 LCC patients.

**Results:** We developed novel TrN and TLODDS classifications for LCC patients that incorporated 7 stages with reference to the AJCC staging system. In comparison to the 8th AJCC TNM and TrN classifications, TLODDS classification demonstrated significantly better discrimination (area under the receiver operating characteristic curve, 0.650 vs. 0.656 vs. 0.661, *p* < 0.001), better model-fitting (Akaike information criteria, 309,287 vs. 308,767 vs. 308,467), and superior net benefits. The predictive performance of the TrN and TLODDS classifications was further verified in the validation and application sets.

**Conclusion:** Both the TrN and TLODDS classifications have better discriminatory ability, model-fitting, and net benefits than the existing TNM classification, and represent an alternative to the current TNM classification for LCC patients.

## Introduction

Colorectal cancer (CRC) is the third most common cancer worldwide and the second most common cause of cancer-specific mortality (1). CRC continues to present challenges to patients and clinicians worldwide. Tumors originating in the distal third of the transverse colon, descending, sigmoid colon, and rectum are classified as left-sided colon cancer (LCC) tumors, whereas tumors originating in the caecum, ascending, and proximal two-thirds of the transverse colon are classified as right-sided colon cancer (RCC) tumors (2, 3). Recent in-depth research on CRC reported differences in clinical manifestations and patient prognosis between LCC and RCC (4–6). In the left-sided tumors, researchers observed chromosomal instability pathway-related mutations, such as KRAS, p53, APC, PIK3CA mutations, and demonstrate polypoid-like morphology (5–8). LCC and RCC have been identified as two different types of solid tumors. Evaluating LCC separately will have a positive impact on the application of individualized treatments to corresponding patients.

An accurate staging system is critical for clinical practice. Furthermore, the status of lymph nodes (N stage) is regarded as the most important predictor of survival (9). The American Joint Committee on Cancer (AJCC) Tumor/Node/Metastasis (TNM) classification is worldwide applied to assess the prognosis of patients with LCC. The American Society of Clinical Oncology and the National Comprehensive Cancer Network have issued guidelines for AJCC N staging of LCC requiring clinical physicians to evaluate at least 12 lymph nodes in clinical work (10). An increase in the number of retrieved lymph nodes (LNs) is known to be associated with a survival benefit for both node-negative and node-positive LCC patients (11). Nevertheless, insufficient lymph node sampling may lead to under-staging and subsequent misestimation of the prognosis (12). We aimed to investigate a more acceptable lymph node staging strategy for LCC, taking into account the quantity of retrieved LNs and metastatic status. We stratified the AJCC N staging based on the lymph node cut-off value and combined subgroups with similar survival rates to establish a revised nodal (rN) staging. Log odds of positive lymph nodes (LODDS) is another potential option as this could be applied to multiple types of cancers (13–15). LODDS has been proved to have a prognostic advantage for CRC (15).

The difference in prognosis between LCC and RCC indicates their different requirements for the staging system. So far, there are no reports exploring the prognostic value of the new staging system for LCC. In this study, we aimed to develop TrN and TLODDS classifications for LCC using a training set acquired from the US Surveillance, Epidemiology, and End Results (SEER) database. We compared the discrimination, model-fitting, and net benefits of the novel classifications to the 8th AJCC TNM classification to determine whether they could improve survival stratification. Finally, we investigated the predictive performance of new models in a validation set from the same database and an application set from a Chinese hospital.

## Materials and methods

### Data sources and screening criteria

48,425 cases of LCC from 2010 to 2015 were identified in the SEER database (National Cancer Institute, https://seer.cancer.gov/) to act as a training set, and 12,436 cases from 2016 to 2017 were considered as a validation set. We determined the codes from the International Classification of Diseases for Oncology for eligible patients: C185, C186, C187, C199 and C209 for LCC patients, and C180, C182, C183 and C184 for RCC patients (3). Data from the SEER database was exempted from informed consent and ethics committee review. We also incorporated an application set of LCC patients from The Third Affiliated Hospital of Sun Yat-sen University (SYSU). The latest follow-up was July 2021. The Institute Ethics Committee approved the study. The flow chart for patient selection is shown in Supplementary Figure S1.

Inclusion criteria: a positive follow-up; the primary tumor was located in the left-sided colon; being AJCC stage I–III.

Exclusion criteria: patients with incomplete clinicopathological information such as numbers of retrieved and positive lymph nodes, TN stage and primary site; patients who died during the first postoperative month.

### Development of TrN and TLODDS staging systems

Lymph node involvement was classified according to the 8th AJCC pathological N (pN) classification (pN0: no metastasis; pN1a: 1 metastatic lymph node; pN1b: 2-3 metastatic LNs; pN1c: no metastatic lymph node, but there are tumor deposits (TD) in subserosal, mesenteric, pericolonic or perirectal tissue; pN2a: 4-6 metastatic LNs; pN2b: 7 and more metastatic LNs) (9). The optimal cut-off value of retrieved LNs in patients with LCC calculated by X-tile software in the training set was 14. Then, we developed a lymph node staging strategy called rN staging to homogenize the nodal classification of LCC cohorts comprising of both >14 (Adequate set) and ≤14 (Inadequate set) LNs. Six pN substages from pN0 to pN2b were obtained by using the 8th AJCC pN stage in Inadequate and Adequate sets, respectively. Table 1 shows the detailed overall survival (OS) rates of the 8th AJCC pN stage. By dividing it into its respective Inadequate set and Adequate set, we found that the 5-year OS rates of N0_inadequate_ to N2a_inadequate_ were closer to that of N1a_adequate_ to N2b_adequate_, rather than their corresponding N0_adequate_ to N2a_adequate_. As displayed in Figure 2, there was no significant difference between Kaplan–Meier OS curves of patients under pN0_inadequate_ and pN1a_adequate_ (*p* = 0.73), pN1a_inadequate_ and pN1c_adequate_ (*p* = 0.59), pN1c_inadequate_ and pN2b_adequate_ (*p* = 0.39). The *p*-value of survival curves of pN1b_inadequate_ and pN2a_adequate_ was less than that of pN1b_inadequate_ and pN1c_adequate_. Table 2 showed that subgroups with similar survival rates were then aggregated to determine the rN stage. Next, we replaced the pN classification of the 8th AJCC pTNM staging system with our rN classification to form the pTrNM classification. Log-rank tests for OS were conducted between two neighboring substages and 19 χ^2^ values were generated (Table 3). The Cox proportional hazards model was used to estimate hazard ratio (HR) values for per substage (using the HR of T1rN0 as a reference), and all substages were sorted by HR values from the lowest (T1rN0) to the highest (T4brN2b). Five peak cutoff χ^2^ values (9.024, 9.294, 9.074, 4.953, 5.479) were identified in LCC patients, and merge substages between neighboring peak cutoff χ^2^ values. But there were far too many substages from substage T2rN0 to T4brN0. We chose substage T1rN2b to be stopped in the middle depending on the survival curve and HR value of each substage (Figure 3A; Table 3). We developed seven categories for the TrN classification (I, IIA, IIB, IIC, IIIA, IIIB, IIIC).

**TABLE 1:** 5-year overall survival (OS) rates of corresponding N0 to N2b of >14 LNs vs. ≤14 LNs cohorts.

The 8th AJCC pN stage	5-Year OS rate (%)	
	The number of LNs retrieved >14 (Adequate set)	The number of LNs retrieved ≤14 (Inadequate set)
pN0	82.0	78.1
pN1a	78.3	70.6
pN1b	73.1	65.1
pN1c	71.0	55.6
pN2a	68.9	57.1
pN2b	53.8	46.1

**TABLE 2:** The rN staging for LCC in the training set according to the 8^th^ AJCC pN classification stratified into Adequate (>14 LNs) and Inadequate (≤14 LNs) set.

The 8th AJCC pN stage	rN stage	
	Adequate set(No.)	Inadequate set (No.)
pN0	rN0 (*n* = 15,989)	rN1a (*n* = 13,338)
pN1a	rN1a (*n* = 3,601)	rN1c(*n* = 2,648)
pN1b	rN1b(*n* = 3,578)	rN2a (*n* = 2,280)
pN1c	rN1c(*n* = 464)	rN2b(*n* = 451)
pN2a	rN2a (*n* = 2,322)	rN2b(*n* = 1,163)
pN2b	rN2b(*n* = 2060)	rN2b(*n* = 531)

**TABLE 3:** The development of the TrN classification for patients with LCC in the training set.

TrN staging system	5-Y OS, % (95% CI)	HR (95% CI)	Log-rank (Mantel-Cox)	
			χ^2^-value	*p*-value
Stage I				
T1rN0	90.2 (89.1–91.3)	1.00(Ref.)	-	-
Stage IIA				
T1rN1	87.8 (86.9–88.7)	1.20 (1.07–1.35)	9.024	0.003
Stage IIB				
T2rN0	86.9 (85.7–88.1)	1.40 (1.24–1.59)	9.294	0.002
T1rN2a	83.8 (79.2–88.8)	1.66 (1.25–2.20)	1.307	0.253
T2rN1	81.8 (80.6–83.0)	1.89 (1.69–2.11)	0.891	0.345
T2rN2a	79.0 (75.3–82.8)	1.92 (1.57–2.35)	0.035	0.852
T3rN0	80.1 (79.2–81.0)	2.05 (1.84–2.27)	0.494	0.482
Stage IIC				
T1rN2b	70.9 (61.7–81.5)	2.59 (1.77–3.80)	1.52	0.218
T3rN1	73.7 (72.9–74.5)	2.68 (2.42–2.97)	0.028	0.867
T2rN2b	71.8 (66.1–77.9)	2.91 (2.30–3.68)	0.52	0.471
T3rN2a	67.9 (66.3–69.6)	3.30 (2.95–3.68)	1.266	0.26
T4arN0	67.3 (63.9–71.0)	3.39 (2.91–3.95)	0.165	0.684
T4brN0	65.1 (61.8–68.5)	3.64 (3.15–4.20)	0.726	0.394
Stage IIIA				
T4arN1	58.5 (55.9–61.2)	4.48 (3.96–5.07)	9.074	0.003
T3rN2b	56.8 (55.0–58.7)	4.76 (4.26–5.30)	1.615	0.204
Stage IIIB				
T4brN1	54.3 (51.2–57.5)	5.33 (4.69–6.05)	4.953	0.026
T4arN2a	53.3 (48.9–58.0)	5.34 (4.58–6.23)	0.003	0.957
Stage IIIC				
T4brN2a	43.8 (37.9–50.6)	6.79 (5.64–8.18)	5.479	0.019
T4arN2b	41.2 (37.6–45.2)	7.84 (6.86–8.96)	2.441	0.118
T4brN2b	36.6 (31.5–42.4)	8.87 (7.52–10.46)	2.108	0.146

LODDS was estimated by: $LODDS=\log\frac{pLNs+0.5}{LNs-pLNs+0.5}$ , where the pLNs is the number of positive lymph nodes and LNs is the total number of retrieved lymph nodes, and 0.5 is added to both numerator and denomination to avoid singularity(16). In the training set, patients with LCC were classified into 20 substages (divided by unit 0.2, as shown in Table 4). HR values were ordered from the lowest (using LODDS ≤ −1.9 as a reference) to the highest (LODDS>1.7). Log-rank tests were conducted between two neighboring LODDS substages and 19 χ^2^ values were generated. Four peaks of χ^2^ values were identified as the cutoff values. Using the four identified cutoff values for LODDS, a novel LODDS stage with five categories was developed (LODDS1≤−1.5; −1.5<LODDS2≤−0.9; −0.9<LODDS3≤−0.3; −0.3<LODDS4≤0.1; LODDS5>0.1). We replaced the pN classification of the 8th AJCC pTNM staging system with our LODDS classification to form the pTLODDSM classification. Log-rank tests for OS were conducted between two neighboring substages and 25 χ^2^ values were generated (Table 5). Six peak cutoff χ^2^ values (16.814, 22.778, 76.608, 7.771, 8.919, 8.414) were identified. We merged substages between neighboring peak cutoff χ^2^ values, so as to obtain seven categories for the TLODDS classification (I, IIA, IIB, IIC, IIIA, IIIB, IIIC).

**TABLE 4:** LODDS staging for patients with LCC in the training set.

LODDS-value	Log-rank (Mantel-Cox)	
	χ^2^-value	*p*-value
≤−1.9	-	-
−1.9∼−1.7	1.576	0.209
−1.7∼−1.5	0.748	0.387
−1.5∼−1.3	**27.887**	**<0.001**
−1.3∼−1.1	4.786	0.029
−1.1∼−0.9	7.783	0.005
−0.9∼−0.7	**12.928**	**<0.001**
−0.7∼−0.5	5.101	0.024
−0.5∼−0.3	1.615	0.204
−0.3∼-0.1	**29.759**	**<0.001**
−0.1∼0.1	5.236	0.022
0.1∼0.3	**7.974**	**0.005**
0.3∼0.5	0.127	0.721
0.5∼0.7	1.405	0.236
0.7∼0.9	2.62	0.106
0.9∼1.1	0.058	0.81
1.1∼1.3	5.808	0.016
1.3∼1.5	0.851	0.356
1.5∼1.7	2.435	0.119
>1.7	0.151	0.697

The chi‐square values in bold represent 4 peaks as the cut‐off values. The *p* values in bold are the *p* values corresponding to the cutoff values.

**TABLE 5:** The development of the TLODDS classification for patients with LCC in the training set.

TLODDS staging system	5-Y OS, % (95% CI)	HR (95% CI)	Log-rank (Mantel-Cox)	
			χ^2^-value	*p*-value
Stage I				
T1LODDS1	91.0 (89.9–92.2)	1.00(Ref.)	-	-
Stage IIA				
T1LODDS2	87.6 (86.6–88.6)	1.31 (1.15–1.49)	16.814	<0.001
T1LODDS3	86.9 (84.8–89.1)	1.46 (1.22–1.75)	1.598	0.206
T2LODDS1	86.8 (85.6–88.1)	1.54 (1.34–1.76)	0.567	0.451
Stage IIB				
T2LODDS2	82.6 (81.4–83.9)	1.98 (1.75–2.24)	22.778	<0.001
T2LODDS3	80.0 (77.7–82.4)	2.13 (1.82–2.49)	1.164	0.281
T3LODDS1	80.5 (79.7–81.4)	2.17 (1.93–2.44)	0.154	0.695
Stage IIC				
T3LODDS2	75.1 (74.2–75.9)	2.77 (2.48–3.11)	76.608	<0.001
T2LODDS4	70.7 (64.1–78.0)	3.12 (2.37–4.10)	0.842	0.359
T1LODDS4	72.4 (62.6–83.8)	3.20 (2.15–4.77)	0.016	0.899
T2LODDS5	66.1 (55.7–78.4)	3.51 (2.39–5.17)	0.076	0.783
T3LODDS3	68.2 (67.0–69.5)	3.58 (3.19–4.02)	0.006	0.937
T1LODDS5	69.7 (57.1–85.0)	3.70 (2.30–5.94)	0.026	0.873
T4aLODDS1	66.3 (62.8–70.0)	3.75 (3.20–4.40)	0	0.999
T4bLODDS1	65.9 (62.6–69.3)	3.91 (3.36–4.56)	0.286	0.593
Stage IIIA				
T4aLODDS2	59.9 (57.0–63.0)	4.79 (4.16–5.51)	7.771	0.005
T3LODDS4	58.3 (55.9–60.8)	4.80 (4.22–5.46)	0.021	0.883
T4aLODDS3	54.4 (51.1–57.9)	5.55 (4.81–6.39)	5.48	0.019
T4bLODDS2	55.0 (51.7–58.6)	5.56 (4.82–6.41)	0.012	0.914
Stage IIIB				
T4aLODDS4	46.7 (41.5–52.6)	7.25 (6.08–8.64)	8.919	0.003
T4bLODDS3	45.3 (40.8–50.4)	7.37 (6.28–8.65)	0.018	0.893
T3LODDS5	44.6 (41.4–48.0)	7.42 (6.48–8.51)	0.037	0.848
T4bLODDS4	40.7 (33.5–49.5)	7.985 (6.361–10.023)	0.361	0.548
Stage IIIC				
T4aLODDS5	30.8 (25.6–37.0)	11.91 (9.99–14.19)	8.414	0.004
T4bLODDS5	26.4 (19.6–35.5)	14.02 (11.20–17.55)	2.455	0.117

### Statistical analysis

We conducted a descriptive analysis of the study population. Continuous variables were expressed as median (interquartile range), while categorical variables were expressed as count and percentage. Their clinical characteristics were compared using Pearson’s χ^2^ test. The Kaplan–Meier method was used to calculate OS, and log-rank tests were used to compare survival differences between the groups. A cox proportional regression hazard model was used to identify risk factors significantly associated with OS. X-Tile software (https://medicine.yale.edu/lab/rimm/research/software.aspx) was used to identify the potential cut-off values for each LN group based on minimal probability (P) values (17). The predictive accuracy of the model was evaluated by the value of area under the receiver operating characteristic curve (AUC). A larger AUC value represented a more accurate model prediction. The linear trend χ^2^ test was used to measure the discriminatory ability and gradient monotonicity. The likelihood ratio χ^2^ test was used to assess the homogeneity (no significant differences in survival among patients with the same stage) within each stage. A higher linear trend chi-square score showed better discriminatory ability and monotonicity, while higher likelihood ratio chi-square scores meant better homogeneity. Akaike information criteria (AIC) and Bayesian information criterion (BIC) represented the model fitting level (18). A smaller AIC or BIC value indicated a more desirable model for prediction of OS outcomes. Clinical benefits were assessed by decision curve analyses (DCAs) (19, 20).

All analyses were performed with the R software (version 4.1.1; R Foundation for Statistical Computing, Vienna, Austria) and SPSS version 22.0 for Windows (IBM Corporation, Chicago, IL, USA), and a two-tailed *p*-value <0.05 was considered statistically significant.

## Result

### Relationships between the number of retrieved LNs and prognosis

Supplementary Table S1 shows the demographic and pathological characteristics of the eligible patients. The median number of retrieved LNs in LCC patients was larger than 12 (16 in the training set, 17 in the validation set, and 17 in the application set), implying that the number of LNs retrieved according to the guidelines was even less.

**FIGURE 1:**
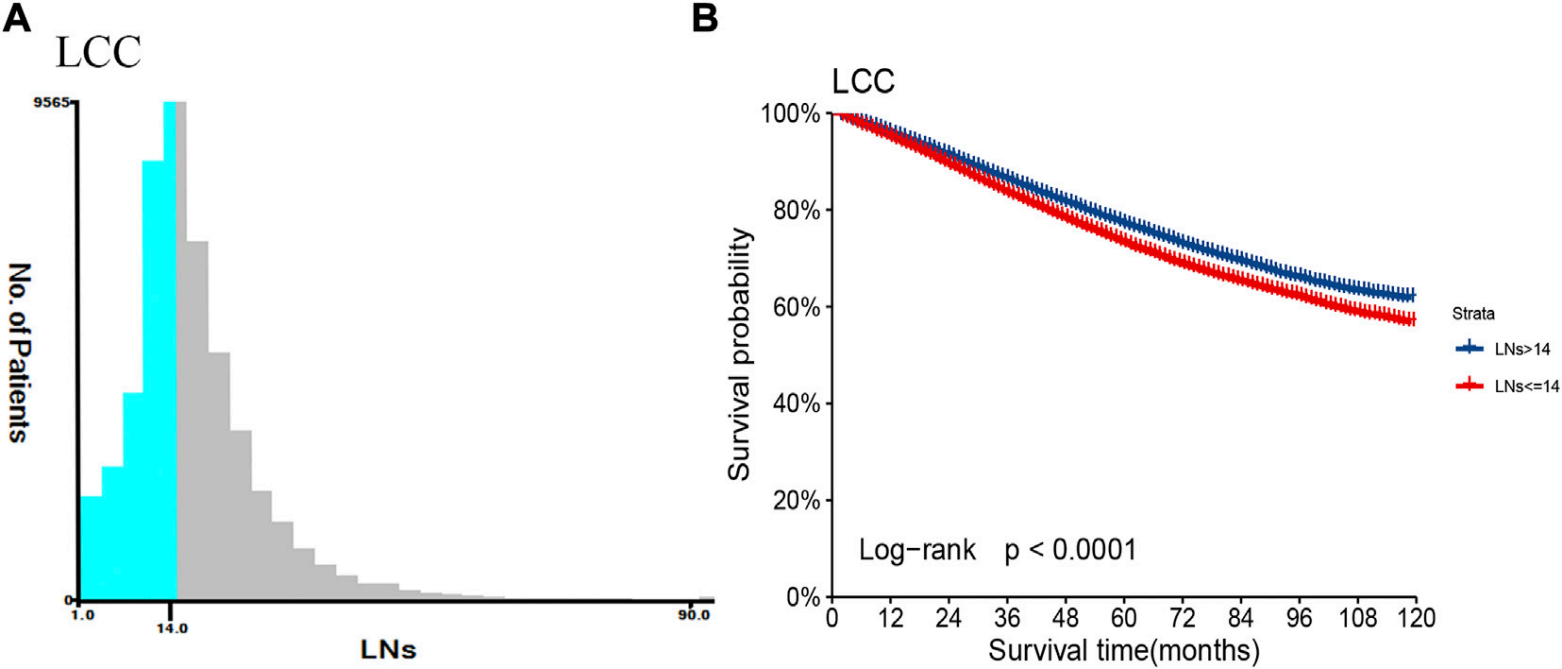
The number of retrieved LNs and prognosis difference between patients with LCC in the training set. **(A)** Based on the results of X-tile software, the best cut-off point of regional lymph node count in patients with LCC was determined. **(B) **The Kaplan-Meier survival curves of patients with LCC were depicted using the retrieved optimal thresholds of the two groups of retrieved LNs.

We used X-tile software to determine the best cut-off value for the number of LNs retrieved by LCC and RCC. According to descriptive statistics, the optimal minimum number of retrieved LNs of RCC is higher than that of LCC (18 vs. 14, as shown in Figure 1; Supplementary Figure S2). It is crucial to develop a lymph node staging system that distinguishes between LCC and RCC. Evaluation of LCC alone can more accurately evaluate the prognosis characteristics of LCC population. By comparing the number of LNs and cut-off value, LCC study population was divided into two categories, and the survival curves of LNs >14 (Adequate set) and LNs ≤14 (Inadequate set) were drawn, respectively. Two subgroups showed significant differences in survival prognosis (*p* < 0.0001, as shown in Figure 1B).

### Development of rN classification and TrN staging system

**FIGURE 2:**
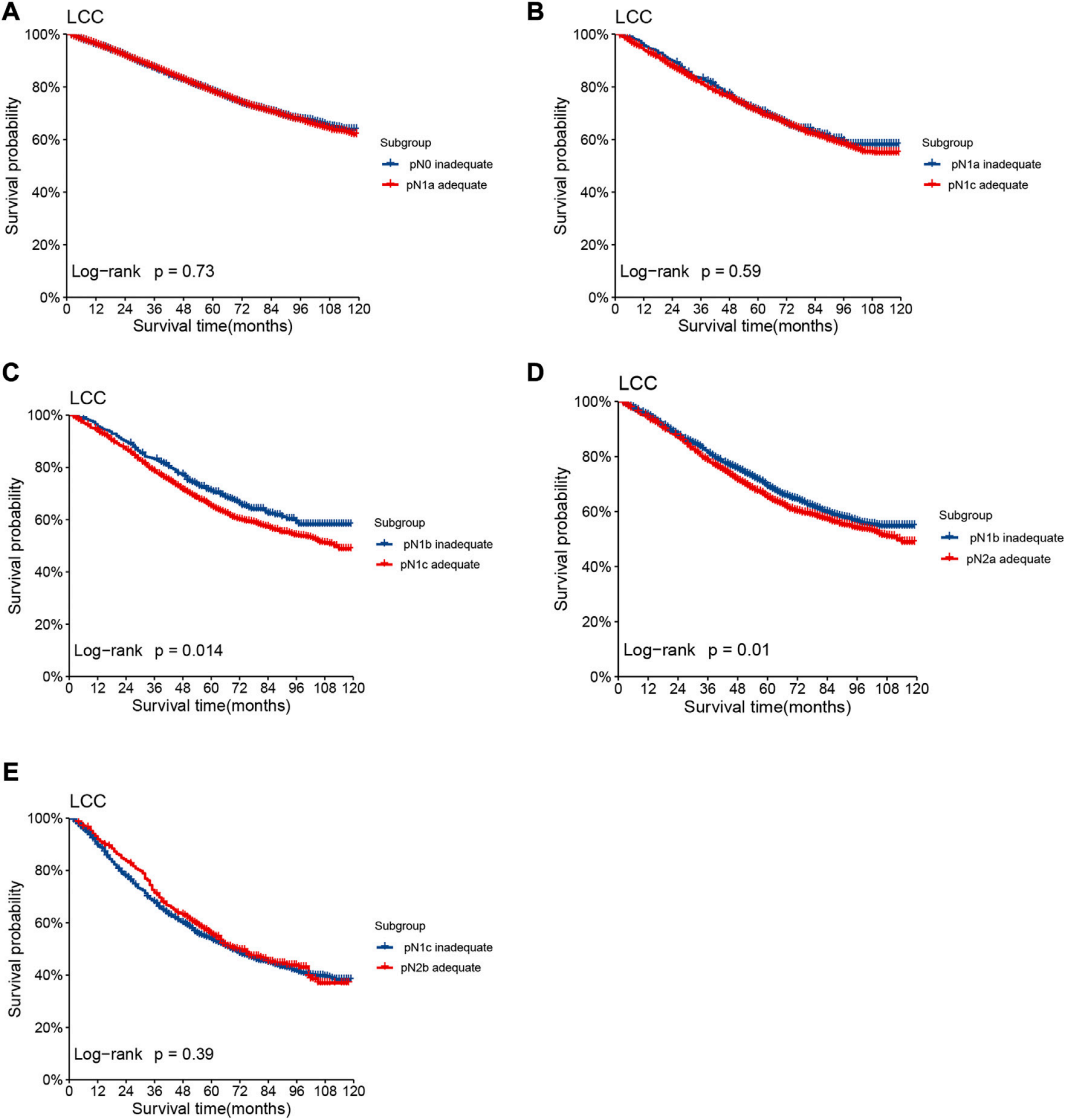
Kaplan–Meier OS curves of patients under different pN stages assigned to Inadequate set and Adequate set. Subgroup **(A)** pN0_inadequate_ vs. pN1a_adequate_, **(B)** pN1a_inadequate_ vs. pN1c_adequate_, **(C)** pN1b_inadequate_ vs. pN1c_adequate_, **(D)** pN1b_inadequate_ vs. pN2a_adequate_, **(E)** pN1c_inadequate_ vs. pN2b_adequate_ of patients with LCC in the training set.

We stratified 6 pN stages in the training set based on the LNs cutoff value and established rN stages by merging subgroups with similar survival rates. The initial six pN subgroups with LNs>14 (Adequate set) were labeled as rN0-rN2b. Subgroups with no significant difference in OS between LNs≤14 (Inadequate set) and LNs>14 (Adequate set) were combined (Figure 2; Table 2).

**FIGURE 3:**
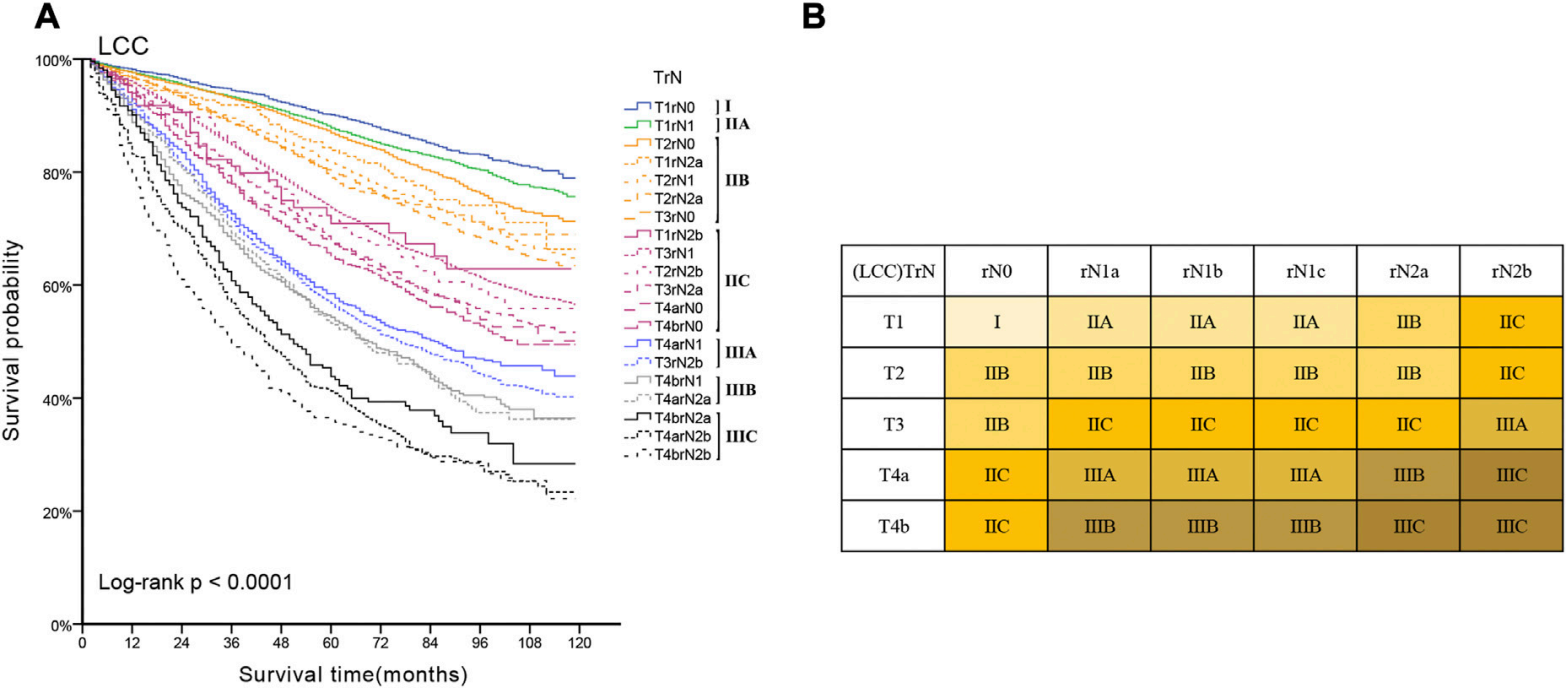
**(A)** Kaplan-Meier estimates for the novel classification of LCC characterized by T-stage and rN stage. **(B)** Details of the novel LCC TrN classification.

Referring to the 8th AJCC TNM stage, the three subgroups of pN1 have no effect on the overall TNM staging results, hence we consolidated rN1a-c into rN1 for subsequent analysis. Five peak cutoff χ^2^ values (9.024, 9.294, 9.074, 4.953, 5.479) were identified in LCC patients. Then we merged substages between neighboring peak cutoff χ^2^ values. A trouble was shown in Table 3. There were far too many substages from substage T2rN0 to T4brN0. We chose substage T1rN2b to be stopped in the middle, depending on the survival curve and HR value of each substage (Figure 3A; Table 3). Twenty substages were clustered into a novel TrN classification of seven clusters (Figure 3B).

### Development of LODDS classification and TLODDS staging system

First, we calculated the LODDS value of each LCC patient in the training set, and then divided the study population into 20 subgroups according to the unit of 0.2. We used the log-rank (Mantel-Cox) test between adjacent subgroups to identify 19 cutoff χ^2^values. We chose four peak cutoff χ^2^ values (27.887, 12.928, 29.759 and 7.974) to distinguish the cut-off values of LODDS (−1.5, −0.9, −0.3 and 0.1) (Table 4). We created a LODDS stage consisting of five categories: (1) LODDS1≤−1.5; (2) −1.5< LODDS2≤−0.9; (3) −0.9< LODDS3≤−0.3; (4) −0.3< LODDS4≤0.1 and (5) LODDS5>0.1.

**FIGURE 4:**
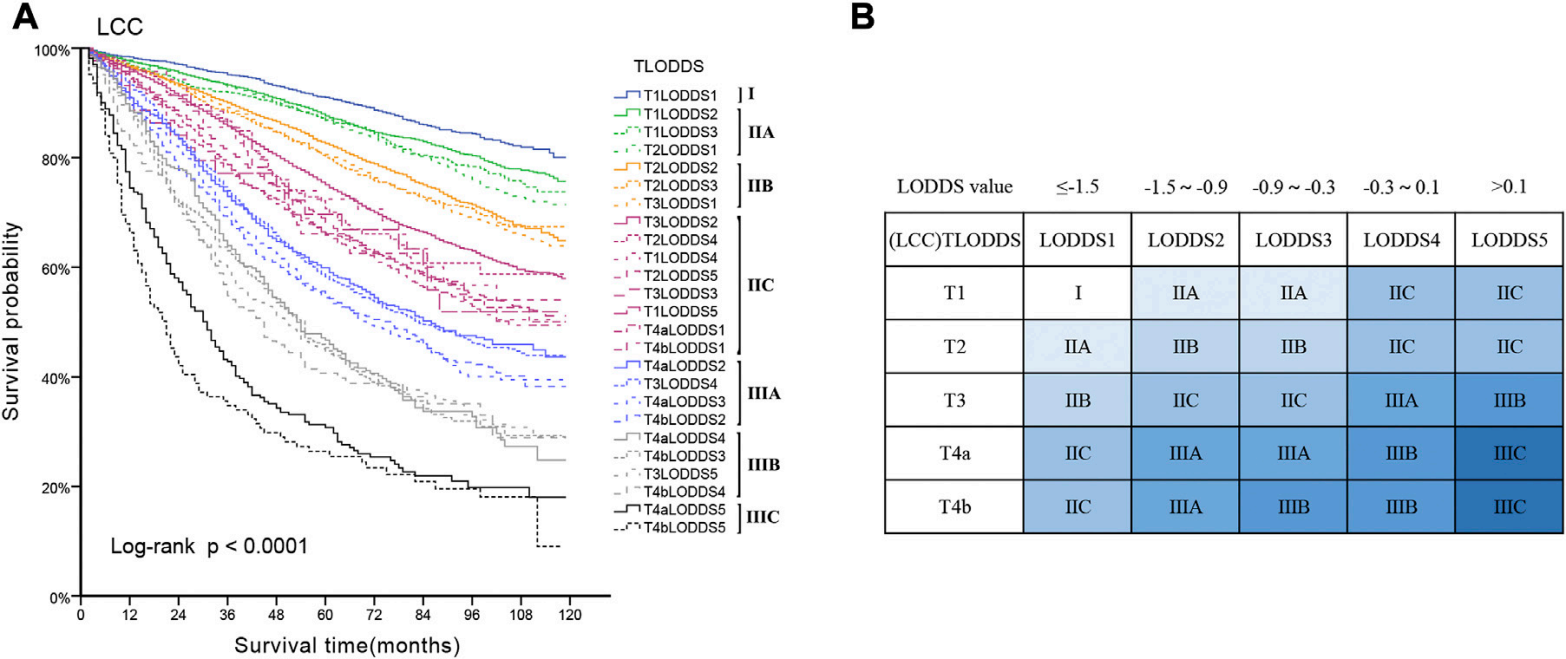
**(A)** Kaplan-Meier estimates for the novel classification of LCC characterized by T-stage and LODDS stage. **(B)** Details of the novel LCC TLODDS classification.

25 substages (T1-4bLODDS1-5) were created by merging the T and LODDS classifications. HR values were ranked from lowest to highest (using T1LODDS1 as a reference, HR = 1.00). We used the log-rank (Mantel-Cox) test between adjacent substages to identify 24 cutoff χ^2^ values. The six peak cutoff χ^2^ values (16.814, 22.778, 76.608, 7.771, 8.919, 8.414) whose corresponding *p* values were less than 0.05 between two neighboring substages were chosen. According to the six peak cutoff χ^2^ values, 25 substages were clustered into a novel TLODDS classification of seven categories (I, IIA, IIB, IIC, IIIA, IIIB, IIIC) (Table 5). Kaplan-Meier curves with log-rank tests and details of the novel TLODDS classification are shown in Figure 4.

### Comparison of the three staging systems

**FIGURE 5:**
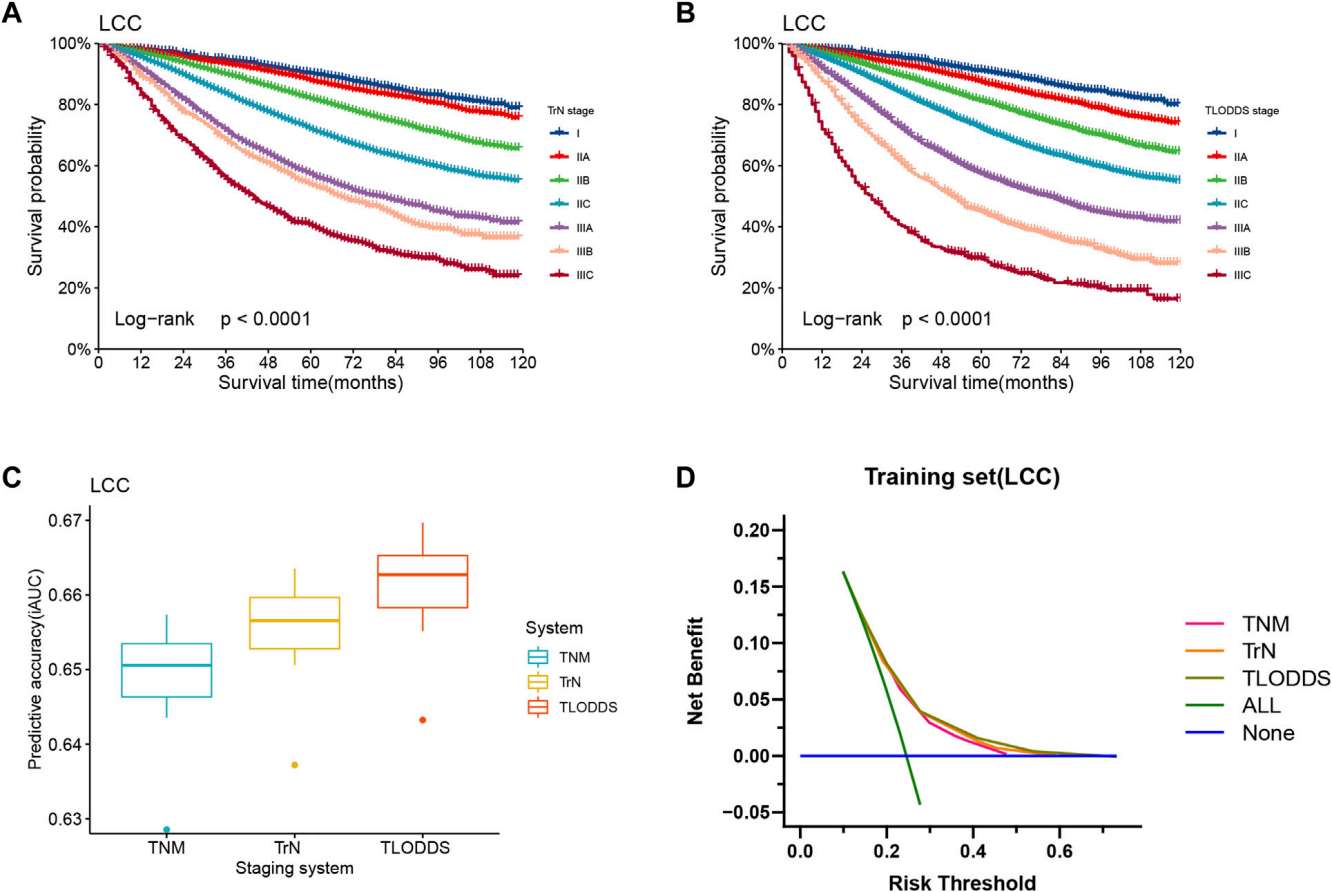
Model evaluation of three staging systems for patients with LCC in the training set. The Kaplan-Meier survival curves of patients with LCC in the training set were depicted according to the **(A)** TrN or **(B)** TLODDS staging system. **(C)** Performance of the TrN and TLODDS staging systems compared with the AJCC TNM staging system. **(D)** Decision curve analyses to compare the estimation of OS among the AJCC TNM, TrN and TLODDS classifications.

In the training set, the 8th AJCC TNM classification displayed an unreasonable predictive performance in that stage IIIA and IIIB had higher 5-year survival rates than stage IIA and IIB (Supplementary Table S2). However, the 5-year OS rates for LCC patients according to the TrN or TLODDS classification showed steadily decreasing trends as the stage increased (5-year OS, stages I to IIIC, 90.2%, 87.8%, 81.9%, 71.9%, 57.3%, 54.0% and 40.6% according to TrN classification; 91.0%, 87.3%, 81.1%, 72.0%, 57.3%, 44.8% and 29.4% according to TLODDS classification). Furthermore, HRs exhibited consistently increasing tendencies as the stage increased (HRs, stages I to IIIC, 1.0, 1.2, 1.9, 2.9, 4.7, 5.4 and 7.9 according to TrN classification; 1.0, 1.4, 2.1, 3.1, 5.1, 7.5 and 12.6 according to TLODDS classification). The TLODDS and TrN classifications also showed better prognostic discrimination (AUC, 0.661 vs. 0.656 vs. 0.650; Liner trend χ^2^ score, 3,104.5 vs. 2,851.6 vs. 1,504.4, *p* < 0.001), better model-fitting (AIC, 308,467 vs. 308,767 vs. 309,287), and superior net benefits than the TNM classification (Figure 5; Table 6).

**TABLE 6:** Comparison of the performance of the 8^th^ AJCC TNM, TrN and TLODDS staging systems in predicting prognosis of LCC.

Staging system	Liner trend χ2	Likelihood ratio χ2	AIC	BIC
Training set				
AJCC TNM	1,504.392(*p* < 0.001)	2,449.929(*p* < 0.001)	309,286.9	309,332.5
TrN	2,851.613(*p* < 0.001)	2,942.981(*p* < 0.001)	308,766.6	308,812.3
TLODDS	3,104.469(*p* < 0.001)	3,212.848(*p* < 0.001)	308,466.7	308,512.4
Validation set				
AJCC TNM	292.980(*p* < 0.001)	475.225(*p* < 0.001)	28,625.5	28,657.8
TrN	590.186(*p* < 0.001)	579.894(*p* < 0.001)	28,512.8	28,545.1
TLODDS	614.707(*p* < 0.001)	621.676(*p* < 0.001)	28,472.0	28,504.3
Application set				
AJCC TNM	85.284(*p* < 0.001)	100.564(*p* < 0.001)	2004.4	2023.2
TrN	92.347(*p* < 0.001)	100.258(*p* < 0.001)	2004.2	2019.9
TLODDS	94.823(*p* < 0.001)	115.049(*p* < 0.001)	1987.5	2003.2

**FIGURE 6:**
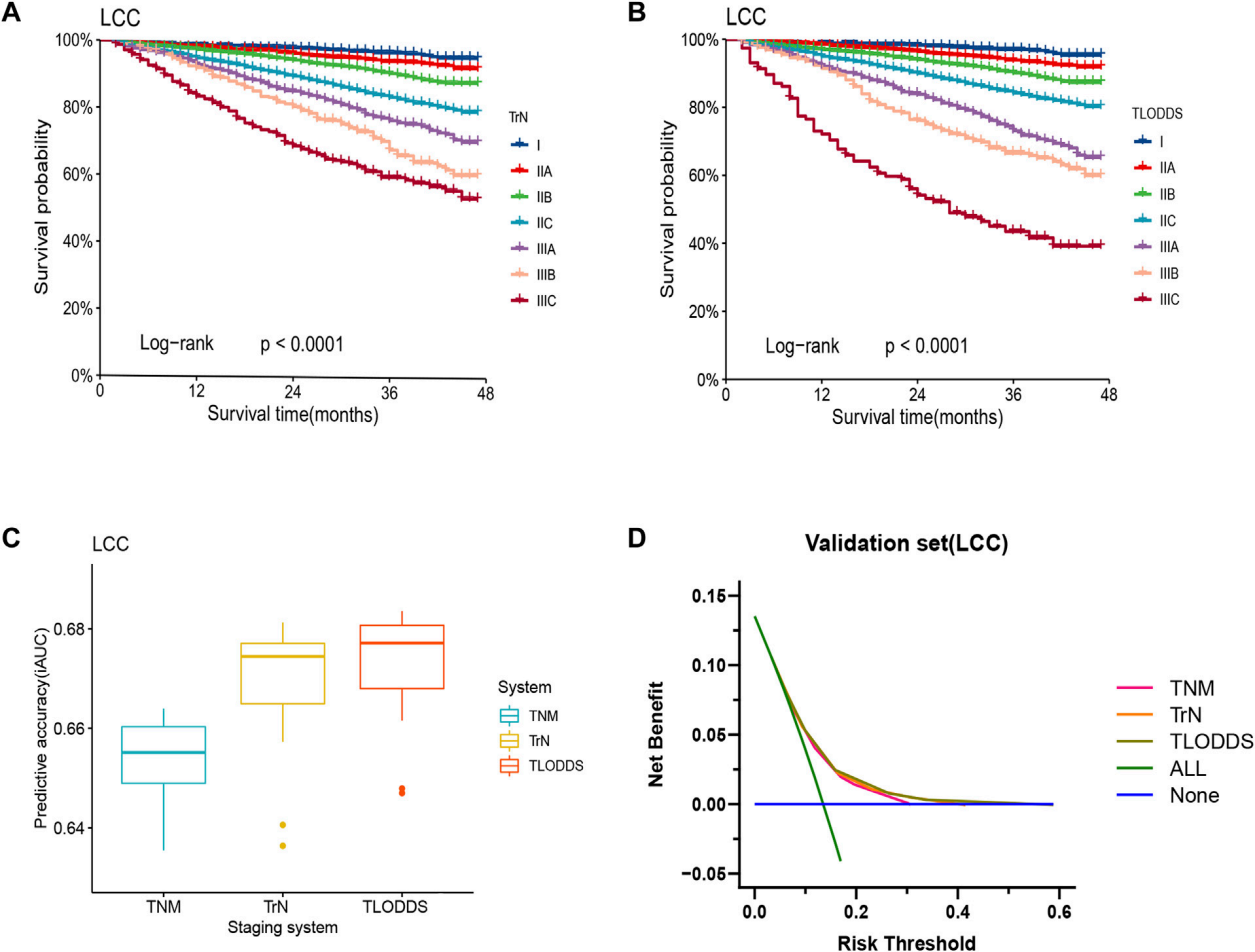
Model evaluation of three staging systems for patients with LCC in the validation set. The Kaplan-Meier survival curves of patients with LCC in the validation set were depicted according to the **(A)** TrN or **(B)** TLODDS staging system. **(C)** Performance of the TrN and TLODDS staging systems compared with the AJCC TNM staging system. **(D)** Decision curve analyses to compare the estimation of OS among the AJCC TNM, TrN and TLODDS classifications.

**FIGURE 7:**
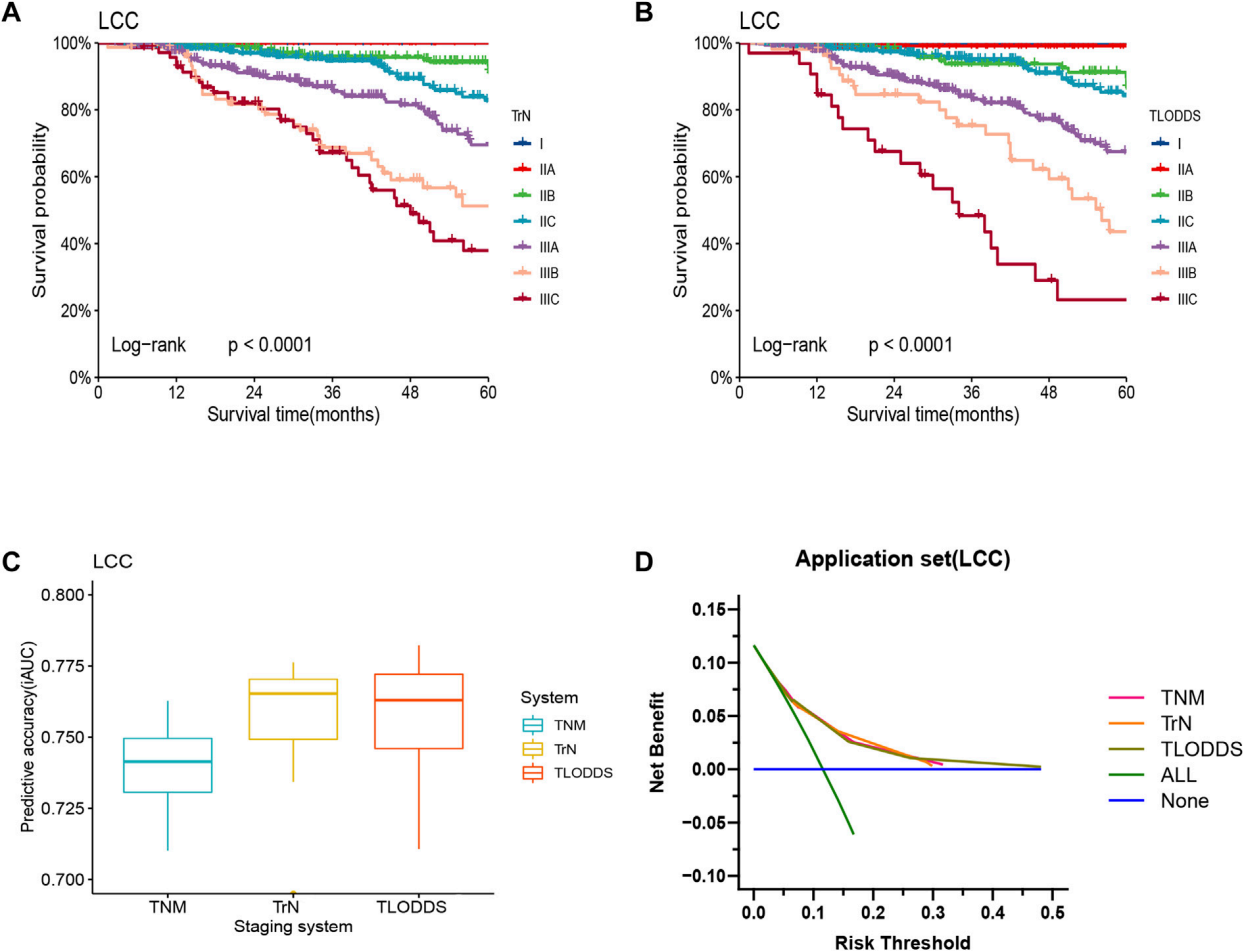
Model evaluation of three staging systems for patients with LCC in the application set. The Kaplan-Meier survival curves of patients with LCC in the application set were depicted according to the **(A)** TrN or **(B)** TLODDS staging system. **(C)** Performance of the TrN and TLODDS staging systems compared with the AJCC TNM staging system. **(D)** Decision curve analyses to compare the estimation of OS among the AJCC TNM, TrN and TLODDS classifications.

The predictive performance of TrN and TLODDS classifications was validated in the validation or application set. As demonstrated in Figures 6, 7, both TrN and TLODDS classifications identify I-IIIC stages effectively in the validation or application set for patients with LCC, with significant differences in OS between neighboring stages. The TLODDS classification showed the best prognosis discrimination across the three staging systems (Figures 6C, 7C), as well as the best model fit and net benefit (Figures 6D, 7D; Table 6).

## Discussion

Tumor staging is a key criterion for evaluating the severity of the cancer, formulating treatment strategies, predicting the recurrence risk and long-term survival of patients. Accurate staging allows cross-sectional comparisons of cohort data from different countries and centers. Currently, stage III of LCC patients even showed a trend of better prognosis than stage II patients according to the 8th AJCC TNM staging system used worldwide. It is crucial to develop new classifications to better stratify LCC survival. The primary flaw of the number-based AJCC pN classification is that the accuracy of the predicted prognosis was significantly influenced by the total number of retrieved LNs (11). The distinct requirements of LCC and RCC patients for the amount of retrieved LNs prompted us to investigate the lymph node staging of the two types of cancer (3). Scholars attempted to use the lymph node ratio (LNR, the ratio of the metastatic LNs and the total retrieved LNs) to predict patient outcomes (21). However, LNR cannot identify individuals without metastatic LNs, and its value is influenced by the overall number of LNs, hence it is not widely used. The recently proposed LODDS has demonstrated high prediction capacity in a variety of cancers (13–15). Andrea et al. and Persiani et al. proved that LODDS classification outperformed AJCC pN and LNR classifications (22, 23).

More researches focused on the prognostic factors of RCC patients while disregarding those of LCC patients. In this study, we developed a TLODDS staging utilizing AJCC T-stage combined with LODDS classification to stratify LCC patients into different survival groups. The TLODDS staging performed better in terms of prognosis discrimination, model fitting, and net benefit. However, the computation of LODDS is relatively complicated, which increases the complexity of clinical application. Furthermore, there are other factors that may affect prognosis, including CEA markers, adjuvant treatments, microsatellite instability, KRAS and BRAF mutation status, etc. (24–26) By integrating these factors, it is possible to further improve our new classification. To successfully apply the TLODDS classification in clinical practice, the development of corresponding clinical calculators and predictive models may be an effective approach. The AJCC pN classification solely takes into account the amount of positive LNs and cannot identify individuals who do not have metastatic LNs. But it is only grouped by counting, which has undoubted advantages in clinical practice. We developed rN classification with the aim of improving prognostic stratification without increasing computational complexity. The rN classification was obtained directly by simple calculations based on postoperative pathology reports. Compared with TLODDS staging, the TrN staging system has the advantages of simpler operation in clinical practice. This suggests that TrN may be a better alternative to the AJCC TNM or TLODDS classification. Both TrN and TLODDS staging systems performed well with regard to distinguishing survival differences among different sub-stages, reflecting their general applicability. These findings were confirmed in the validation set.

However, this study has some shortcomings. The SEER 22 enrollment data represents only 28% of the U.S. population, and these data were not randomly selected, making the analysis biased (27). Additionally, there was an unavoidable unknown bias owing to the retrospective experimental design. Validation in large, multi-center, prospective clinical trials is needed to obtain more reliable results.

## Conclusion

In conclusion, the TrN and TLODDS staging systems can effectively distinguish the survival differences, and more accurately predict the 5-year OS rate of patients undergoing coloproctectomy with insufficient numbers of retrieved LNs compared with that of the TNM staging system.

## Data Availability

The original contributions presented in the study are included in the article/Supplementary Material, further inquiries can be directed to the corresponding authors.
