# Supplementary material for: New staging systems for left-sided colon cancer based on the number of retrieved and metastatic lymph nodes provide a more accurate prognosis
Source: Pathol Oncol Res. 2023 Feb 24;29:1610874. doi: 10.3389/pore.2023.1610874 (PMC9998476; doi:10.3389/pore.2023.1610874)
Supplement: Supplementary file 1 [file DataSheet1.docx]

Supplementary Material


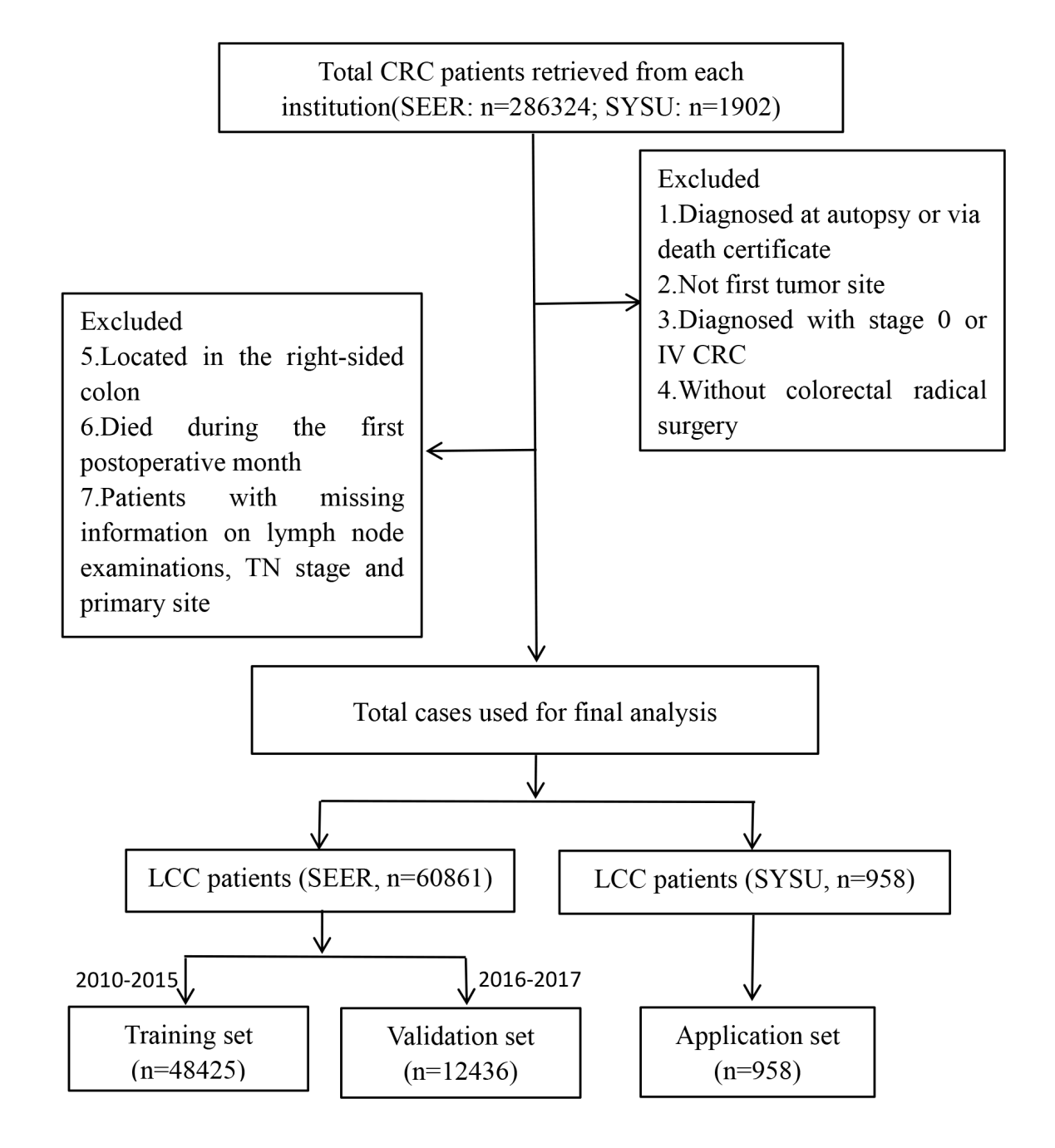


**Supplementary Figure 1.** Flow chart for patient selection.


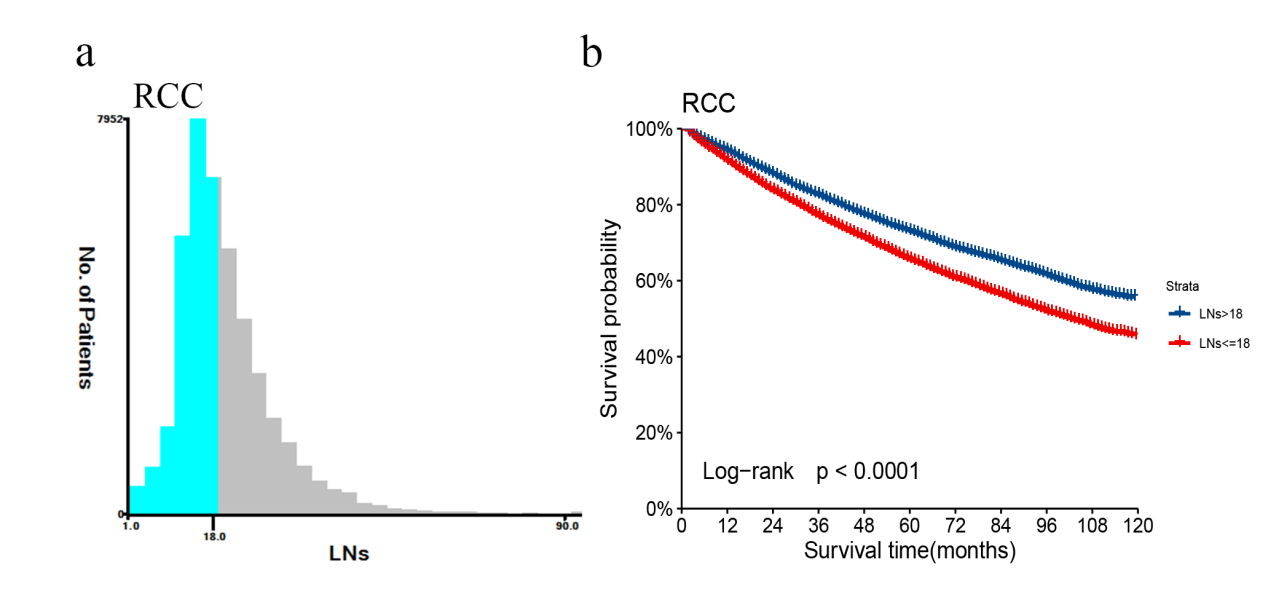


**Supplementary Figure 2.** The number of retrieved LNs and prognosis difference between patients with RCC in the training set. (a) Based on the results of X-tile software, the best cut-off point of regional lymph node count in patients with RCC was determined. (b)The Kaplan-Meier survival curves of patients with RCC were depicted using the retrieved optimal thresholds of the two groups of retrieved LNs.

**Supplementary Table 1.** Demographic and pathological characteristics of study population.

| Characteristics | Training set | Validation set | Application set |
| --- | --- | --- | --- |
|  | N = 48425 (100%) | N=12436(100%) | N=958(100%) |
| Sex (%) |  |  |  |
| Male | 27151 (56.07) | 6878 (55.31) | 612 (63.88) |
| Female | 21274 (43.93) | 5558 (44.69) | 346 (36.12) |
| Age (%) |  |  |  |
| <50 | 7393 (15.27) | 1831 (14.72) | 158 (16.49) |
| 50-69 | 26511 (54.75) | 7018 (56.43) | 561 (58.56) |
| >69 | 14521 (29.99) | 3587 (28.84) | 239 (24.95) |
| Year (%) |  |  |  |
| 2010 | 8275 (17.09) | / | / |
| 2011 | 8150 (16.83) | / | / |
| 2012 | 8190 (16.91) | / | / |
| 2013 | 7723 (15.95) | / | / |
| 2014 | 8151 (16.83) | / | / |
| 2015 | 7936 (16.39) | / | / |
| 2016 | / | 6333 (50.92) | / |
| 2017 | / | 6103 (49.08) | / |
| Race (%) |  |  |  |
| White | 37627 (77.70) | 9402 (75.60) | / |
| Black | 4612 (9.52) | 1213 (9.75) | / |
| Other | 5982 (12.35) | 1678 (13.49) | 958(100) |
| Unknown | 204 (0.42) | 143 (1.15) | / |
| Grade (%) |  |  |  |
| I | 3997 (8.25) | 1153 (9.27) | 19 (1.98) |
| II | 36235 (74.83) | 9543 (76.74) | 872 (91.02) |
| III | 5117 (10.57) | 1159 (9.32) | 62 (6.47) |
| IV | 855 (1.77) | 253 (2.03) | 1 (0.10) |
| Unknown | 2221 (4.59) | 328 (2.64) | 4 (0.42) |
| 8th AJCC T stage(%) |  |  |  |
| T1 | 8210 (16.95) | 2248 (18.08) | 64 (6.68) |
| T2 | 8212 (16.96) | 2287 (18.39) | 183 (19.10) |
| T3 | 26376 (54.47) | 6164 (49.57) | 118 (12.32) |
| T4a | 3241 (6.69) | 1173 (9.43) | 556 (58.04) |
| T4b | 2386 (4.93) | 564 (4.54) | 37 (3.86) |
| 8th AJCC N stage(%) |  |  |  |
| N0 | 29327 (60.56) | 7540 (60.63) | 615 (64.20) |
| N1a | 6249 (12.90) | 1390 (11.18) | 127 (13.26) |
| N1b | 5858 (12.10) | 1443 (11.60) | 110 (11.48) |
| N1c | 915 (1.89) | 490 (3.94) | 2 (0.21) |
| N2a | 3485 (7.20) | 908 (7.30) | 55 (5.74) |
| N2b | 2591 (5.35) | 665 (5.35) | 49 (5.11) |
| 8th AJCC TNM stage(%) |  |  |  |
| I | 13179 (27.22) | 3713 (29.86) | 220 (22.96) |
| IIA | 13705 (28.30) | 3145 (25.29) | 84 (8.77) |
| IIB | 1171 (2.42) | 408 (3.28) | 298 (31.11) |
| IIC | 1264 (2.61) | 273 (2.20) | 13 (1.36) |
| IIIA | 2831 (5.85) | 716 (5.76) | 23 (2.40) |
| IIIB | 12441 (25.69) | 3146 (25.30) | 213 (22.23) |
| IIIC | 3834 (7.92) | 1035 (8.32) | 107 (11.17) |
| Tumor size (%) |  |  |  |
| ＜5cm | 28190 (58.21) | 7609 (61.19) | 677 (70.67) |
| ≥5cm | 15657 (32.33) | 4115 (33.09) | 266 (27.77) |
| Unknown | 4578 (9.45) | 712 (5.73) | 15 (1.57) |
| LNs (median [IQR]) | 16 [12, 21] | 17 [13, 23] | 17 [12, 24] |
| pLNs (median [IQR]) | 0 [0, 1] | 0 [0, 1] | 0 [0, 1] |
| LNR (median [IQR]) | 0.00 [0.00, 0.08] | 0.00 [0.00, 0.08] | 0.00 [0.00, 0.08] |
| LODDS (median [IQR]) | -1.4 [-1.5, -0.8] | -1.4 [-1.6, -0.9] | -1.4 [-1.6, -0.9] |
| Median follow-up (months) | 68 [50, 91] | 33 [27, 40] | 41 [21, 69] |

**Supplementary Table 2.** Survival comparisons among the 8^th^ AJCC TNM, TrN and TLODDS classifications in the training, validation and application sets.

| Stage | AJCC TNM | | TrN | | TLODDS | |
| --- | --- | --- | --- | --- | --- | --- |
|  | 5-Y OS (95%CI) | HR | 5-Y OS (95%CI) | HR | 5-Y OS (95%CI) | HR |
| Training set |  |  |  |  |  |  |
| I | 86.6(86.0-87.2) | 1.0(Ref.) | 90.2(89.1-91.3) | 1.0(Ref.) | 91.0(89.9-92.2) | 1.0(Ref.) |
| IIA | 77.4(76.6-78.1) | 1.7 | 87.8(86.9-88.7) | 1.2 | 87.3(86.5-88.0) | 1.4 |
| IIB | 63.5(60.7-66.4) | 2.8 | 81.9(81.3-82.5) | 1.9 | 81.1(80.4-81.8) | 2.1 |
| IIC | 60.5(57.8-63.3) | 3.1 | 71.9(71.3-72.6) | 2.9 | 72.0(71.3-72.7) | 3.1 |
| IIIA | 83.9(82.5-85.3) | 1.1 | 57.3(55.8-58.9) | 4.7 | 57.3(55.8-58.8) | 5.1 |
| IIIB | 69.5(68.7-70.3) | 2.3 | 54.0(51.5-56.6) | 5.4 | 44.8(42.5-47.2) | 7.5 |
| IIIC | 51.3(49.7-53.0) | 4.1 | 40.6(37.9-43.5) | 7.9 | 29.4(25.2-34.4) | 12.6 |
| Validation set | 3-Y OS(95%CI) |  | 3-Y OS(95%CI) |  | 3-Y OS(95%CI) |  |
| I | 93.5(92.6-94.3) | 1.0(Ref.) | 96.9(95.8-98.1) | 1.0(Ref.) | 97.0(95.8-98.3) | 1.0(Ref.) |
| IIA | 88.3(87.1-89.6) | 1.8 | 94.4(93.0-95.8) | 1.8 | 93.9(92.8-95.0) | 1.9 |
| IIB | 80.9(76.9-85.2) | 3.2 | 90.8(89.8-91.7) | 3.0 | 90.1(89.0-91.2) | 3.1 |
| IIC | 68.0(61.9-74.8) | 5.2 | 83.7(82.4-85.0) | 5.4 | 84.2(82.9-85.4) | 5.1 |
| IIIA | 93.1(91.1-95.2) | 1.0 | 76.7(74.1-79.5) | 7.9 | 73.1(70.3-76.0) | 9.2 |
| IIIB | 82.6(81.1-84.1) | 2.7 | 67.9(62.9-73.4) | 11.1 | 66.5(61.7-71.7) | 12.2 |
| IIIC | 69.8(66.8-72.9) | 5.3 | 59.8(54.6-65.5) | 16.2 | 43.5(34.7-54.4) | 26.4 |
| Application set | 5-Y OS(95%CI) |  | 5-Y OS(95%CI) |  | 5-Y OS(95%CI) |  |
| I | 95.0(91.0-99.2) | 1.0(Ref.) | 100(100-100) | - | 100(100-100) | - |
| IIA | 81.4(71.1-93.1) | 3.1 | 100(100-100) | 1.0(Ref.) | 99.1(97.3-100.0) | 1.0(Ref.) |
| IIB | 82.9(77.2-89.0) | 2.5 | 91.5(87.1-96.2) | 1.3 | 86.7(79.8-94.3) | 5.3 |
| IIC | 69.8(46.0-100.0) | 5.0 | 82.8(76.6-89.6) | 2.0 | 84.2(78.1-90.9) | 5.1 |
| IIIA | 93.3(81.5-100.0) | 1.8 | 69.5(61.9-78.1) | 4.3 | 67.5(60.9-74.9) | 13.4 |
| IIIB | 64.3(55.9-74.0) | 5.9 | 51.3(39.3-66.9) | 9.6 | 43.6(30.1-63.2) | 23.4 |
| IIIC | 40.8(30.7-54.1) | 12.3 | 37.9(26.0-55.4) | 10.0 | 23.2(10.7-50.3) | 50.5 |
